# Improving Skin-to-Skin Care Among Stable Infants in a Level III NICU: A Quality Improvement Project Using Kotter’s Change Management Model

**DOI:** 10.7759/cureus.87735

**Published:** 2025-07-11

**Authors:** Isaac Lati, Vivian Carrasquilla-Lopez, Bianca Chakravorty, Alok Bhutada, Mehbeen Khan

**Affiliations:** 1 Department of Neonatology, State University of New York Downstate Health Sciences University, Brooklyn, USA; 2 Department of Neonatology, Maimonides Medical Center, Brooklyn, USA; 3 School of Nursing, York College, City University of New York, Jamaica, USA; 4 Department of Pediatrics, Maimonides Medical Center, Brooklyn, USA

**Keywords:** change management, culture shift, kangaroo mother care (kmc), neonatal intensive care unit (nicu), northeast united states, pdsa cycle, quality improvement (qi), skin-to-skin care

## Abstract

Introduction

Skin-to-skin care (SSC), also known as kangaroo care, is defined as placing an infant directly prone, typically with the infant's chest against the caregiver's chest. SSC has demonstrated significant benefits for newborns, including improved thermoregulation, cardiorespiratory stability, reduced stress responses, and enhanced neurodevelopmental outcomes. While its use is widely promoted for preterm or critically ill infants, term and clinically stable infants are often overlooked in SSC initiatives despite evidence suggesting they, too, obtain substantial benefit. This is especially true in neonatal intensive care units (NICUs). Most quality improvement (QI) studies related to SSC revolve around critically ill preterm infants. Efforts to improve SSC rates tend to prioritize acutely ill infants, with less attention given to those with short-term NICU admissions and stable conditions. Therefore, non-acutely ill infants miss out on the benefits of SSC. This QI project aimed to address this disparity.

Setting

The initiative was conducted in a level III NICU located in an urban hospital in Brooklyn, New York.

Methods

Using the Plan-Do-Study-Act (PDSA) model and Kotter's Eight-Step Change Management framework, we sought to achieve a Specific, Measurable, Achievable, Relevant, Time-Bound (SMART) aim: to increase SSC to 80% of non-acutely ill infants >35 weeks gestational age, admitted to the NICU without respiratory support by hospital day 2, within one year. Key interventions included electronic medical record (EMR) updates, nurse education, parent engagement, and visual bedside cues.

Results

Between December 2023 and April 2024, 277 newborns admitted to the NICU met our study criteria. SSC rates improved from a baseline of 63.8% to 80.5% across three PDSA cycles. A four-month follow-up phase demonstrated sustained compliance (80.5%, n = 103), indicating durable change.

Conclusion

Non-acutely ill infants are underserved by the benefit of SSC due to institutional culture and resource allocation that emphasize care for high-acuity patients. This QI initiative shows that, when applied intentionally, structured change management can effectively integrate SSC into routine care for this overlooked population. Through structured change management, we included SSC into routine NICU care, achieving and sustaining our goal.

## Introduction

Skin-to-skin care (SSC), also known as kangaroo care, is the practice of placing a newborn in direct contact with a parent’s bare chest, promoting warmth, bonding, and physiologic stability [1]. In preterm infants, SSC has been shown to improve neurodevelopmental outcomes, stabilize blood glucose, enhance respiratory function, reduce pain, support better sleep, and promote weight gain [1-9]. It has also been associated with increased breastmilk production and greater breastfeeding success [7]. Given these well-established benefits in preterm infants, it is reasonable to extrapolate that a similar benefit exists in term and clinically stable infants from SSC.

Despite broad endorsements for preterm or acutely ill infants, term and stable neonates are often excluded from SSC initiatives, ranging from individual problems to systems-based issues [3,6]. Parents frequently perceive obstacles such as the fear of injuring the infant, challenges related to the environment, available space, and a lack of education about the benefits and practice of SSC [3,10]. Nursing providers may also encounter barriers, including discomfort with physically transporting the neonate between the isolette and the caregiver’s chest [4]. Additional constraints include time limitations, staff shortages, and restricted parental visitation. Furthermore, non-acutely ill infants are often paired in a higher nurse-to-patient ratio assignment, exacerbating time constraints. This culture of caring for non-critically ill patients in the NICU sheds light on the need for changes as it relates to delivering SSC. Change management techniques previously developed and used in the corporate setting can be applied to the NICU to instill culture change to improve systems-based practices [11,12].

This quality improvement (QI) initiative aimed to improve SSC rates among non-acutely ill, late preterm, or term infants in a level II NICU using Kotter's change management model and PDSA framework.

## Materials and methods

Setting and population

This QI initiative was conducted at Maimonides Medical Center in Brooklyn, New York, over eight months. The NICU is classified as a level III unit and, at the time of the study, had a capacity of 31 beds. The unit typically maintains an average daily census of 25 to 30 neonates. The hospital’s primary workforce includes neonatologists, fellows, resident physicians, neonatal nurse practitioners, registered nurses, and patient care associates. Additionally, the unit operates as a teaching facility, with medical students regularly completing rotations in the unit. Most of the bedside procedures within the unit are performed by fellows, residents, and nurse practitioners under the supervision of attending neonatologists, collectively referred to as NICU staff in the study.

Study design

**Figure 1:**
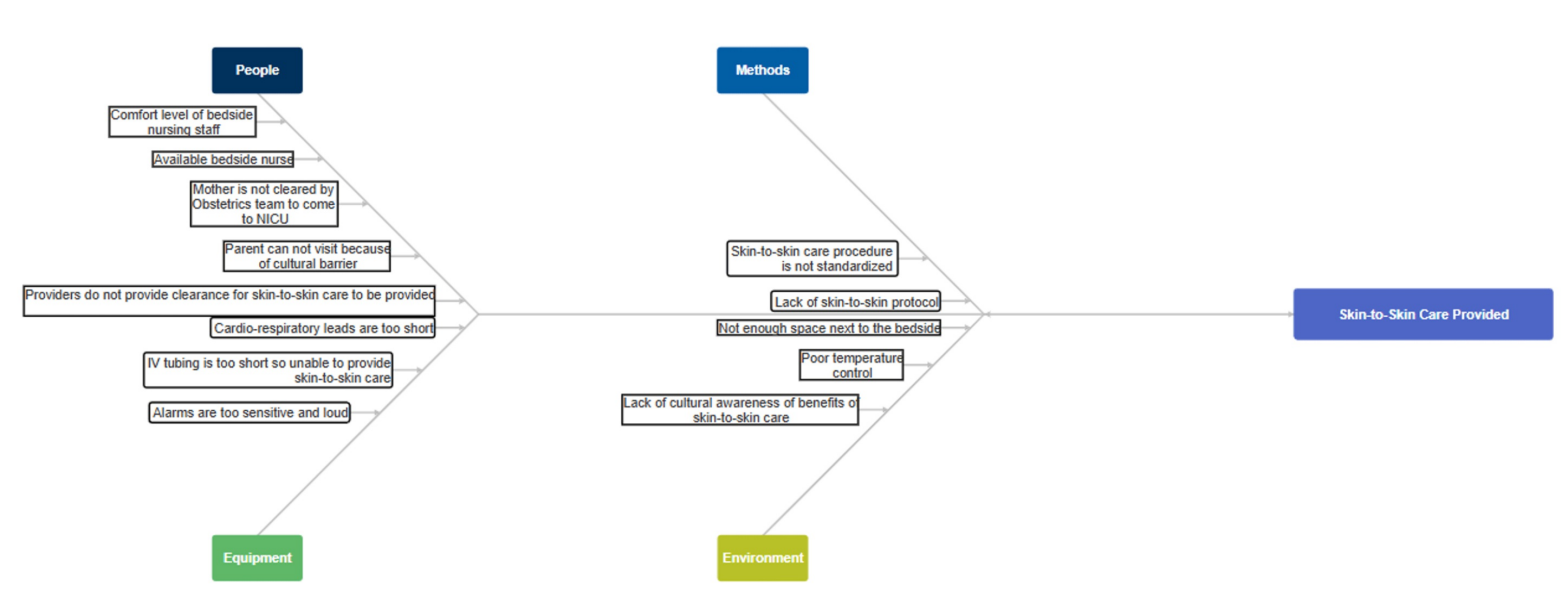
Fishbone diagram

The Plan-Do-Study-Act (PDSA) Methodology for QI was employed, and a Specific, Measurable, Achievable, Relevant, Time-Bound (SMART) aim: to achieve SSC by hospital day 2 in 80% of non-acutely ill infants (>35 weeks, room air, stable) within one year. The QI team consisted of attending neonatologists, a fellow, a nurse practitioner, nurse managers, and nurse champions. An interdisciplinary team of stakeholders and advisers was utilized to guide the planning and implementation of the QI initiative. A fishbone diagram was developed (Figure 1), and a detailed description of the PDSA cycles used is listed below.

Skin-to-skin care in the context of this study was defined using the World Health Organization and American Academy of Pediatrics definition “practice of placing infants in direct contact with their mothers or caregivers with the ventral skin of the infant facing and touching the ventral side of the mother/caregiver's chest” [10].

Infants were included in the study if they were non-acutely ill, ≥ 35 weeks gestational age (GA), on room air, hemodynamically stable, and had short-term admission indications (e.g., hypoglycemia, transient tachypnea).

Infants who were on continuous positive airway pressure (CPAP), ventilators, with umbilical catheters, or under phototherapy within 48 hours of life were excluded from the study. 

This QI project received Institutional Review Board (IRB) exempt status (ID 20221210mmc) with a waiver of informed consent, justified by minimal risk, the use of de-identified data, and implementation of standard care practices.

Our SMART aim was to increase the percentage of eligible infants receiving SSC by hospital day 2 to ≥80% within one year. 

Design and framework

We employed the PDSA model and Kotter’s Eight-Step Change Management theory. A fishbone diagram was used to identify barriers across domains: staffing, environment, policy, documentation, and caregiver beliefs. These insights shaped interventions.

Pre-intervention phase

Baseline data were collected before the start of the study in order to assess the need for change implementation. A total of 47 infants were included in this baseline analysis. A survey was then sent to the bedside nursing staff to assess the perceived barriers of SSC.

Intervention phase

The first PDSA cycle was conducted between December 5, 2023, and January 6, 2024; the second PDSA cycle was conducted between January 8, 2024, and February 7, 2024; and the third PDSA cycle was conducted between February 8, 2024, and April 17, 2024.

Post-intervention phase

A QA cycle was done to ensure maintenance of the SMART aim. This analysis examined data four months following completion of the study.

Interventions

Since there were no existing policies or protocols regarding SSC in our unit, change management techniques were employed to foster a culture shift. To identify barriers within the unit, survey responses from bedside nurses during the pre-intervention phase of the study guided the selection of implementable changes. The lack of a reliable mode of documentation was identified as a major barrier to understanding why SSC was not being provided.

During the first PDSA cycle (December 2023-January 2024), the primary intervention involved updating the electronic medical record (EMR) with support from the information systems department. The documentation was revised to capture not just whether SSC occurred, but also the reasons it may not have been offered. New options included “SSC provided,” “parents not at bedside,” “parent declined,” and “not offered,” with the latter including a free-text field for nurses to document specific reasons. Commonly cited barriers included respiratory distress requiring CPAP, phototherapy for jaundice, and the presence of an umbilical catheter. Following this intervention, SSC rates increased to 74.6%.

During the second PDSA cycle (January-February 2024), data collected from the first cycle were analyzed to identify common reasons why SSC was not being provided. These insights informed a targeted education campaign, led by nurse champions, aimed at addressing misconceptions and nursing biases related to SSC eligibility. Nurses continued to use the free-text documentation feature to record specific barriers to SSC, which increased awareness but also led to more cautious decision-making. As a result, SSC rates declined to 65.3% during this cycle.

During the third PDSA cycle (February-April 2024), staff provided parental education on the benefits of SSC to increase engagement and awareness. Providers also began formally discussing SSC eligibility during daily rounds, allowing barriers to be identified and addressed in real time. The inclusion of parents in these discussions further emphasized the importance of SSC. To reinforce the initiative, a visual aid - a magnet placed near the isolette - was used to indicate that the infant was cleared and ready for SSC. This served as a prompt for both staff and parents to initiate SSC. As a result of these combined interventions, SSC rates increased to 80.5%, surpassing the SMART aim.

Data collection process

Data on admissions to the NICU during the specified time frames were obtained from the electronic medical records. To determine whether skin-to-skin contact was completed, individual charts were reviewed. Throughout the data collection process, we maintained discretion and ensured anonymity to comply with the Health Insurance Portability and Accountability Act of 1996 (HIPAA) standards. The data were stored on local computers as well as in a shared folder within the organization.

Measures

Process Measure

The process measure that was used is the percentage of infants who received SSC by hospital day 2.

Outcome Measure

The primary outcome measure was an increase in the percentage of infants who received SSC by hospital day 2 pre-intervention and post-intervention.

Balancing Measure

The balancing measure, which ensures that there is no unintended negative consequence as a result of the study, was to ensure that there is no perceived increased workload on bedside nursing staff as a result of the study’s implementation.

Statistical analysis

As a QI study, we used descriptive statistics to track changes in SSC compliance rates over time. The primary outcome was the percentage of eligible infants who received SSC by hospital day 2. No inferential statistical tests (e.g., p-values or confidence intervals) were applied, in alignment with the Institute for Healthcare Improvement (IHI) methodology, which prioritizes practical, real-time evaluation over hypothesis testing.

GA and birth weight data for infants included in each PDSA cycle are summarized as follows: During the baseline (pre-intervention) phase, the mean GA was 38.0 weeks (range 35.3-40.2; SD ±1.0), and the mean weight was 3.16 kg (range 2.4-4.0; SD ±0.32). In PDSA1, the mean GA was 38.0 weeks (range 35.2-40.4; SD ±1.1), and the mean weight was 3.22 kg (range 2.5-4.1; SD ±0.35). In PDSA2, the mean GA was 37.6 weeks (range 35.0-40.1; SD ±1.0), and the mean weight was 3.08 kg (range 2.3-4.0; SD ±0.34). In PDSA3, the mean GA was 38.0 weeks (range 35.4-40.5; SD ±1.0), and the mean weight was 3.16 kg (range 2.5-4.2; SD ±0.36). During the quality assurance (QA) phase, no new interventions were introduced. A total of 103 infants were monitored, and SSC compliance remained stable at 80.5% (n = 103).

This consistency, despite staffing and census fluctuations, indicates a lasting culture shift, not dependent on active enforcement.

These descriptive data demonstrate consistency in the patient population across all cycles, supporting the interpretation that observed changes in SSC rates were primarily attributable to the interventions rather than demographic variability.

## Results

The baseline data collected were an observational study of 47 infants with an average GA of 38 weeks and an average weight of 3.16 kg. Following the implementation of PDSA1, a total of 79 infants were included, with an average GA of 38 weeks and an average weight of 3.22 kg. This intervention increased the percentage of infants receiving SSC by hospital day 2, rising from a baseline rate of 63.8% (n = 47) to 74.6% (n = 79). However, the subsequent implementation of PDSA2, which included 75 infants with an average GA of 37 weeks and four days and an average weight of 3.08 kg, demonstrated a decrease in SSC rates to 65.3% (n = 75). This decline is likely attributable to a change in documentation protocols that allowed nurses to provide reasons for not performing SSC, potentially prompting a more selective application of the intervention.

**Figure 2:**
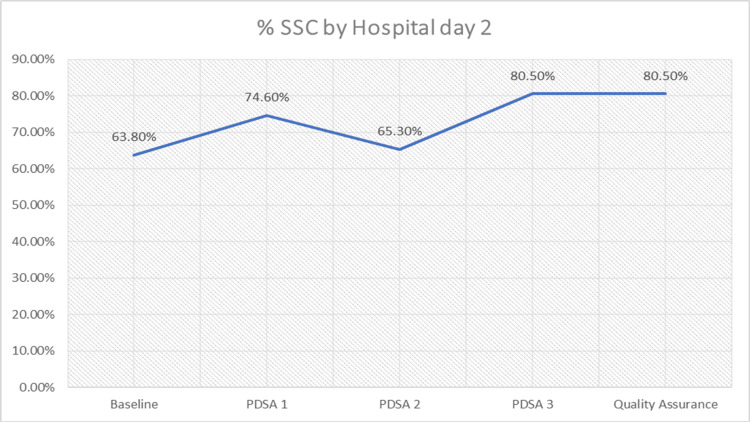
PDSA cycle and percent compliance This figure shows non-linear improvement: initial gains (PDSA1); drop during PDSA2 due to detailed documentation; strong rise post-education (PDSA3); sustained success in quality assurance phase. Takeaway: Documentation alone is insufficient; staff education and culture change are critical for lasting success. PDSA, Plan-Do-Study-Act; SSC, skin-to-skin care

The PDSA3 intervention, which included 123 infants with an average GA of 38 weeks and an average weight of 3.16 kg, yielded an increase in SSC rates to 80.5% (n = 123), reaching the established goal. To ensure continued compliance, an additional quality assurance cycle was conducted, involving infants with an average GA of 38 weeks and two days and an average weight of 3.16 kg, which maintained the SSC rate at 80.5% (n = 103) (Figure 2).

## Discussion

This QI initiative successfully increased the rates of SSC by day of life 2 in infants born at 35 weeks of gestation and above. By employing the PDSA model, we systematically addressed barriers to SSC in our NICU, with each cycle building on the insights and challenges observed in previous phases. The results indicate that targeted education and structured guidelines can effectively address barriers to SSC implementation, even for babies who may not traditionally be viewed as needing intensive interventions, and can instill lasting culture change within the unit.

Implementation of change management techniques to achieve goal

**Figure 3:**
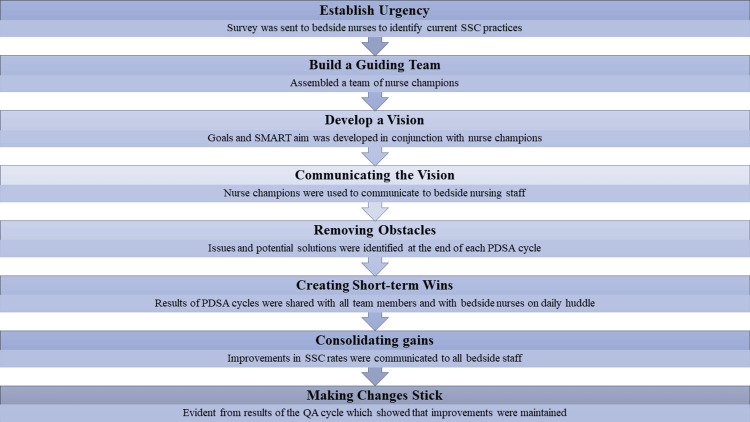
Interventions targeted to change model Adopted from the model described in Reference [12].

John Kotter’s stages of change model is one of the most widely utilized frameworks for managing organizational change and has been effectively adapted for use both in healthcare management and in the corporate setting [11,12]. By leveraging techniques from Kotter’s model, we were able to achieve our goals (Figure 3).

According to Kotter, there are eight steps essential for achieving transformational change [11,12]. The first step, establishing urgency, was addressed by sending out a survey to nurses to identify their current SSC practices [11]. This approach was effective in increasing urgency, as it prompted nursing staff to communicate with each other and critically reflect on their practices, leading to an increase in SSC rates following the PDSA cycle, despite minimal changes in documentation practices. This is one of the few NICU QI studies targeting stable term infants for SSC, a population often neglected. The structured use of Kotter’s model enabled sustainable change beyond one-time interventions. Staff involvement through nursing champions promoted buy-in and adaptation.

Building a guiding team, the second step in Kotter’s model, involved forming a team of nurse champions who were responsible for educating their peers about SSC contraindications and procedures [11]. These nurse champions were selected based on their willingness and commitment to instituting change. Adding a free-text EMR field in PDSA2 revealed common barriers like phototherapy and IV access. Although SSC rates dipped, this phase informed targeted education and policy clarification in PDSA3. The third step, developing a vision or strategy, was articulated early in the project through a SMART aim [11]. Aligning the goal with organizational values, we shifted the focus to the benefits of SSC for infants, making the vision more relatable to the nurses. Visual “SSC-ready” magnets and structured discussions during rounds created ongoing cues for staff and empowered parents. These tactics helped normalize SSC as standard care.

Communicating the vision, the fourth step was facilitated by the nurse champions who acted as intermediaries between the bedside nursing staff and researchers [11]. They relayed issues and potential solutions, while discussions during rounds about whether a baby was cleared for SSC further emphasized the importance of SSC to bedside staff. Steps 5, 6, and 7 - removing obstacles, creating short-term wins, and consolidating gains - were mediated by the nurse champions, who addressed issues in real-time with bedside staff members [11]. After each PDSA cycle, results were shared with nurse champions and then communicated to bedside staff, which likely motivated continued SSC implementation.

Finally, the eighth step, making changes stick, was assessed through a QA cycle, which confirmed the persistence of the implemented changes [11]. Discussing the importance of SSC with staff and encouraging SSC daily significantly improved SSC rates. The four-month follow-up showed SSC rates remained at 80.5% without additional interventions. This suggests the culture shift was embedded, not dependent on enforcement.

Impact of documentation

One of the key findings of this project is the critical role that accurate and transparent documentation plays in improving SSC rates. A retrospective study performed in 2022 highlighted the importance of accurate documentation as it relates to quality care, which is why we chose this intervention to improve SSC rates [13]. During the first PDSA cycle, allowing nurses to document reasons for not offering SSC proved to be an essential step. This process brought SSC to the forefront of care decisions and created an opportunity for the care team to reflect on and address specific challenges in providing SSC. This increased awareness among nursing staff likely contributed to the observed rise in SSC rates despite minimal intervention. We believe that changes in documentation practices align with stage six of Kotter’s stages of change, as bedside nurses can experience “wins” when identifying barriers to SSC, empowering them to make decisions.

Addressing barriers and misperceptions

The second PDSA cycle, which introduced a blank box for nurses to fill in specific reasons for not offering SSC, revealed several barriers. These included medical conditions such as the need for phototherapy, issues with IV access, and episodes of instability in the infants. Although SSC rates temporarily dipped during this phase, the information was invaluable for understanding the specific reasons why SSC was not being provided. This allowed nurse champions to address these barriers. This step correlates to stage six of Kotter’s model - removing obstacles [11,12]. The education and support provided were essential in reversing the dip in SSC rates and ensuring that eligible babies receive SSC.

Sustaining improvement through visual aids

The fourth and final PDSA cycle introduced additional strategies, such as clearing babies for SSC during rounds and using visual aids (magnets placed at the bedside) to remind staff of SSC eligibility. These interventions provided a constant visual cue, reinforcing the importance of SSC and ensuring that it remained a priority in daily care. This contributed to achieving and sustaining the goal of 80% SSC by day of life 2. "Clearing" the baby on rounds to receive SSC opened the floor for discussion among providers and bedside nurses, allowing for further collaboration and discussion on whether SSC could be offered to a specific baby. We also found that placing the magnets bedside served as a reminder to parents to request permission to perform SSC. This strategy relates to the last step in Kotter’s model for change in that it helped the changes stick.

Nurse buy-in and culture shift

A recurring theme throughout the project was the importance of nurse buy-in. As described above in Kotter's Model, buy-in may be used to establish urgency [11,12]. When examining other studies on the importance of nurse buy-in, the importance of this concept allows for increased ownership and engagement among bedside nursing staff [14]. As the primary caregivers in the NICU, nurses play a pivotal role in implementing and promoting SSC. By involving them in the decision-making process and providing opportunities to document and discuss barriers, we fostered a sense of ownership over SSC outcomes. This engagement likely contributed to the overall success of the initiative.

Moreover, this project highlights the importance of creating a supportive environment that encourages SSC as part of routine care. The use of visual aids and consistent feedback helped shift the NICU culture towards one where SSC is seen as the standard of care for eligible infants, both term and stable, as well as preterm and ill babies.

Lewin's change model

Another change model described by Kurt Lewin can potentially be utilized to instill institutional change. This model focuses on three main steps: unfreezing, change, and refreezing [14-16].

Unfreezing involves discussing the need for change with the aim of breaking down the existing status quo [15,16]. This step typically includes identifying what needs to change, understanding why the change is necessary, and engaging stakeholders in discussions about the change [15]. This phase can be met with resistance from group members as the current status quo is challenged, which may be uncomfortable for some group members [16].

The change step is where administrators implement the discussed changes, such as instituting new policies, behaviors, and interventions, while providing support and resources to help individuals adapt [15,16]. During this phase, effective communication strategies and stakeholder involvement are crucial to ensure success [16].

The refreezing phase is arguably the most important, as it aims to embed the changes into the new culture [15,16]. Like the previous step, effective communication to identify and address new barriers is essential for sustained success [16].

This model was not selected for use in our study because its relatively simplistic structure does not support a gradual, incremental change process, a feature that we felt was necessary to address the long-term culture already established in our NICU and minimize disruption, as identified during our stakeholder meetings.

Study strengths and limitations

This QI initiative demonstrated that structured change management, when combined with frontline engagement, can effectively increase SSC rates among a historically overlooked NICU population, clinically stable term infants. A key strength of the study was its intentional focus on this neglected group, which is often excluded from SSC initiatives that primarily target critically ill or preterm neonates. The use of Kotter’s Eight-Step Change Management model provided a structured framework to guide behavioral and cultural transformation within the unit. Interventions were co-developed with staff, incorporating real-time feedback and qualitative input, which strengthened feasibility and engagement. Notably, the sustained improvement observed in the QA phase four months post-intervention supports the durability of the change.

Qualitative insights, such as documentation notes and staff feedback, were instrumental in identifying misconceptions during PDSA2, which explained the temporary decline in SSC rates and guided the successful corrective actions in PDSA3.

However, this study has several limitations. As a single-center project conducted in an urban NICU in Brooklyn, New York, findings may not be generalizable to other settings with different patient demographics, staffing structures, or institutional cultures. In addition, potential selection bias may have influenced which infants were deemed eligible for SSC, as bedside providers determined readiness based on clinical judgment. The use of descriptive statistics, in line with QI methodology, means inferential analysis was not performed, limiting the ability to assess statistical significance or causality. Moreover, unmeasured confounding factors, such as day-to-day variations in staffing, patient acuity, or parental presence, may have influenced SSC rates. The follow-up period was limited to four months. 

This project focused exclusively on infants ≥35 weeks of gestation on room air to first establish reliable SSC practices among more stable patients. A second phase, targeting more fragile infants requiring respiratory or intensive support, is planned based on these foundational results. While this study centered on Kotter’s change management model, other frameworks may also be effective in eliciting cultural change in healthcare environments. Ultimately, this work offers a replicable structure for other NICUs, with the understanding that local adaptation is essential for successful implementation.

## Conclusions

By applying Kotter’s Eight-Step Change Management model, we systematically and sustainably improved SSC practices in our NICU. This structured approach to organizational change enabled a meaningful culture shift, leading to a significant increase in SSC rates, from a baseline of 63.8% to 80.5%, which was maintained in the post-intervention phase. The success of this QI initiative highlights the value of combining system-level interventions with targeted cultural and educational strategies. Notably, this study is among the few in the healthcare setting to apply Kotter’s framework to drive cultural transformation in neonatal care. It offers a replicable roadmap for other NICUs aiming to promote SSC equity, particularly for clinically stable term infants who are often overlooked in traditional SSC initiatives.

This work reinforces a key principle in healthcare improvement: sustainable change requires more than revised protocols - it demands intentional culture building, real-time data feedback, and active frontline engagement. Looking ahead, our next focus will be on maintaining these gains and expanding SSC efforts to include more fragile and medically complex infants.
